# The effect of Chinese medicine therapeutics on HIV/AIDS: a systematic review and network meta-analysis

**DOI:** 10.3389/frph.2025.1689063

**Published:** 2025-11-04

**Authors:** Wu Hongxi, Huang Qinglian, Liu Yiyang, Liang Jiale, Huang Zhenjin, Luo Huiping, Zhang Rongxin, Wang Ruting, Song Yuanbo, Jiang Feng

**Affiliations:** ^1^ Graduate School, Guangxi University of Chinese Medicine, Nanning, Guangxi, China; ^2^ Affiliated Hospital, Guangdong Medical University, Zhanjiang, Guangdong, China; ^3^ Ruikang Hospital, Guangxi University of Chinese Medicine, Nanning, Guangxi, China

**Keywords:** Chinese medicine, AIDS, HIV, ART, network meta-analysis

## Abstract

**Background:**

Although antiretroviral therapy (ART) effectively suppresses HIV, incomplete immune reconstitution affects 20%–30% of adherent patients. Chinese Medicine (CM) demonstrates potential as a complementary therapy for human immunodeficiency virus (HIV)/acquired immune deficiency syndrome (AIDS), yet its long-term impact on immune recovery remains unestablished. This network meta-analysis (NMA) aimed to compare CM interventions for enhancing CD4^+^ T-cell counts and overall efficacy in HIV/AIDS management.

**Methods:**

We systematically searched PubMed, Embase, Web of Science, and the Cochrane Library from inception to 27 August 2024 for randomized controlled trials (RCTs) and observational studies on CM for HIV/AIDS. Bayesian NMA was conducted using R 4.2.2 with BUGSnet 1.1.0 package. Surface under cumulative ranking (SUCRA) probabilities ranked interventions. Risk of bias was assessed with Cochrane ROB 2.0 for RCTs and Newcastle-Ottawa Scale for observational studies (PROSPERO: CRD42024560340).

**Results:**

A total of 34 studies (*n* = 8,933 participants) evaluating 16 interventions were included. Key findings: For CD4^+^ restoration, Chinese herbal formulae plus ART significantly outperformed ART alone (MD = 163 cells/μL, 95% Bayesian credible interval [CrI]: 3.93–326.46), ranking first (SUCRA = 0.92). Single herbs plus ART ranked second for CD4^+^ recovery (MD = 178.54, 95% CrI: −188.57–553.24; SUCRA = 0.85). In overall treatment efficacy (survival/quality of life), Chinese herbal formulae plus Western medical therapy demonstrated the highest SUCRA (0.96).

**Conclusion:**

CM-ART combinations—particularly Chinese herbal formulae with ART—optimize immune reconstitution in HIV/AIDS. Chinese herbal formulae plus ART represents the most effective CD4^+^ restoration strategy. These findings support integrating evidence-based CM into HIV care, but pharmacokinetic interactions and long-term safety require validation through multicenter trials.

**Systematic review registration:**

https://www.crd.york.ac.uk/prospero/display_record.php?ID=CRD42024560340, PROSPERO CRD42024560340.

## Graphical Abstract

**Figure:**
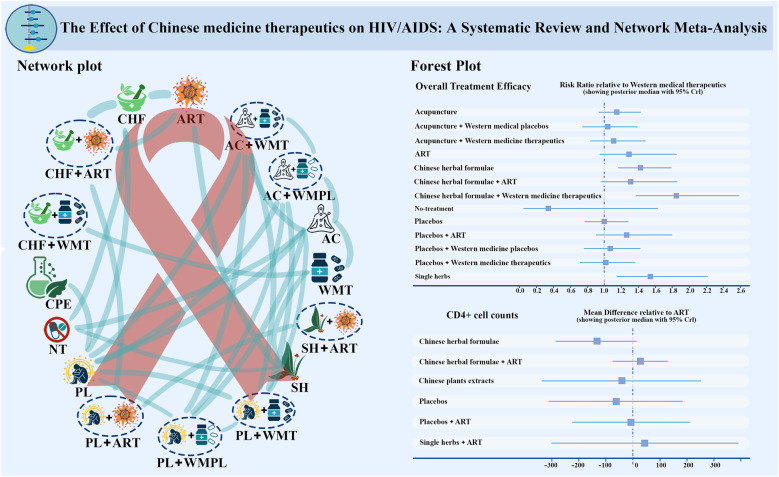
Network and forest plots evaluated the efficacy of TCM in treating HIV/AIDS. The network diagram illustrated direct comparisons between different therapeutic regimens. The two forest plots respectively compared Western medicine versus other therapies in terms of overall response rate, and ART versus other treatments regarding changes in CD4+ cell count.

## 1 Background

Human immunodeficiency virus (HIV) is a retrovirus that attacks the immune system, leading to a complex disease known as acquired immune deficiency syndrome (AIDS) (1, 2). This viral infection and its resultant disease continue to pose a significant global health challenge (3). Over the past 50 years, the predominant strain of HIV—human immunodeficiency virus type 1—has caused a worldwide pandemic (4).

In 2024, there were approximately 40.8 million individuals living with HIV/AIDS worldwide, with approximately 31.6 million people receiving antiretroviral therapy (ART) to manage the infection (5). In 2015, the World Health Organization revised its recommendations and advised initiating ART for all HIV-infected individuals, irrespective of their CD4^+^ T lymphocyte levels (6), which plays a crucial role in the immune response.

China reported its first AIDS-related death in 1985 (7) and has since implemented a series of policies to prevent and control the spread of the disease (8). These initiatives have yielded some positive outcomes. However, due to the vast territory and large population of China, the reported number of HIV cases remains relatively high (9, 10). Between 2015 and 2022, Chinese voluntary counselling and testing clinics conducted a total of 22,075,386 HIV tests, resulting in 260,353 newly reported cases (11). China, a country representing one-fifth of the world's population, has a rich history of traditional Chinese Medicine (CM) that spans thousands of years. Chinese Medicine (CM) has been proven to be effective in treating various infectious diseases (12). A recent example of its effectiveness is its role in managing the novel coronavirus disease 2019 caused by the severe acute respiratory syndrome coronavirus 2 virus (13). In China and South Africa, thousands of HIV patients have already undergone treatment with CM, demonstrating that the positive impact of traditional medicine on alleviating symptoms and improving patient-reported outcomes is not merely coincidental (12). CM therapeutics encompasses a range of therapeutic modalities, including Chinese herbal formulae, single herbs, Chinese plant extracts (CPE), acupuncture, moxibustion, and Chinese massage (tuina). These modalities are generally regarded as one kind of complementary therapeutic modality. The utilization of Chinese herbal formulae, single herbs, Chinese plant extracts, acupuncture, moxibustion, and tuina is predicated on the tenets of CM.

The core tenets of CM are its “holistic concept” and the principle of “treatment based on syndrome differentiation.” Guided by these principles, clinical practice often employs personalized combination therapies—such as Chinese herbal formulae, acupuncture, moxibustion, and tuina—to achieve optimal therapeutic effects through synergism (14–16). Consequently, although these interventions differ in their specific modalities and mechanisms of action, they share a unified theoretical foundation and a common therapeutic goal: to restore the balance of Yin-Yang, Qi-Blood, and the Zang-Fu organs (17). Based on this theoretical framework, this review aims to evaluate the existing evidence for various CM-based interventions in HIV management from an integrative perspective. This approach reflects the reality of their clinical application and provides a preliminary basis for developing future comprehensive treatment strategies.

While ART is an effective means of suppressing HIV, it does not eradicate the disease. Those living with HIV/AIDS must take medication for an extended period, potentially for the remainder of their lives (18). CM therapeutics has been employed to treat infectious diseases for millennia. Previous studies have demonstrated that CM therapeutics can increase CD4^+^ T-cell counts and improve the immunological function of patients with HIV/AIDS, with a particular focus on its promotion of patients' immune reconstitution (19–21). Moreover, numerous CM therapies have been demonstrated to alleviate discomfort symptoms that may arise after ART treatment (22–26). However, the majority of these studies were small in scale, with low sample sizes and short observation periods. Consequently, there is a paucity of data on the long-term trends in CD4+ T-cell counts among patients with HIV/AIDS receiving CM therapeutics. In light of the above, the present study aims to conduct a systematic review and network meta-analysis (NMA) of the literature on CM therapy for HIV/AIDS. The objective of this study is to explore the combined use of CM therapeutics and ART, with the aim of leveraging the strengths of both traditional Chinese medicine and Western medicine. The findings of this study are intended to provide evidence-based guidance for the clinical management of HIV/AIDS.

## 2 Methods

We performed this systematic review with network meta-analysis according to a prespecified protocol registered on PROSPERO (CRD42024560340). We followed guidance from PRISMA (27) and the Cochrane Handbook for Systematic Reviews of Interventions (28).

### 2.1 Inclusion and exclusion criteria

#### 2.1.1 Study design

In our analysis, we included randomized controlled trials and several high-quality observational studies that examined the effectiveness of traditional Chinese medicine therapies in the treatment of HIV/AIDS.

### 2.2 Inclusion criteria

#### 2.2.1 Type of participants

The diagnostic criteria for HIV/AIDS were in accordance with the Chinese guidelines for diagnosis and treatment of HIV/AIDS (2021 edition) (29) and the Consensus of Integrative Medicine treatment experts on poor reconstruction of HIV immune function (30). Participants were eligible for inclusion if they had a confirmed HIV-positive status and were receiving care at participating healthcare facilities. Notably, the study population encompassed patients below the age of 18 years, acknowledging the importance of pediatric HIV/AIDS management. Inclusion was not limited by age, gender, or ethnicity, reflecting the diverse demographic affected by the disease. The study aimed to capture a broad spectrum of HIV/AIDS patients to ensure the generalizability of the findings.

#### 2.2.2 Type of interventions

CM therapeutics, including herbal formulae, single herbs, plant extracts, acupuncture, moxibustion, and tuina, is recognized as a complementary treatment approach. Herbal formulae are synergistic combinations of various medicinal substances aimed at boosting CD4^+^ T-cell counts and enhancing immune function in HIV/AIDS patients. Single herbs or extracts, derived from individual medicinal sources, serve to increase CD4^+^ T-cell numbers and provide therapeutic benefits to AIDS patients. Acupuncture, moxibustion, and tuina are techniques that activate acupuncture points through needle insertion, heat application, physical manipulation, electrical stimulation, or drug injection. Integrated CM–Western therapeutic modalities are also employed, all grounded in fundamental TCM principles.

#### 2.2.3 Type of comparisons

In this study, the control group is managed with modern medicine treatments, such as non-ART Western pharmaceuticals (Western medicine therapeutics), ART, or undergoes placebo treatment or no treatment. The treatment group is categorized based on therapeutic strategy: individuals receiving monotherapy with CM; those in a combined treatment arm where CM is integrated with Western medicine, illustrated by the concurrent application of Chinese herbal formulae with ART; and those undergoing a comprehensive CM regimen, potentially including diverse practices like acupuncture and massage, to explore their respective impacts on health outcomes.

#### 2.2.4 Type of outcome indicators

Outcome measures for this study include the following:

• CD4 and CD8 cell counts: Reflect the engagement of the immune system and viral control in HIV-infected individuals (31, 32).

• HIV viral load (33, 34): A biomarker of viral activity and treatment response.

• Survival rate (35, 36): Indicates longevity and success of disease management.

• Quality of life (QoL) assessments (37, 38): Using tools such as the Medical Outcome Study-HIV Health Survey (MOS-HIV), Functional Assessment of Human Immunodeficiency Virus Infection (FAHI), Functional Assessment of Chronic IllnessTherapy (FACIT-Sp), World Health Organization Quality of Life Questionnaire for HIV brief version (WHOQOL-HIV BREF) (39) to assess overall well-being and life satisfaction.

• Anthropometric measures: Body weight and body mass index (BMI) to monitor nutritional status and health risks (40, 41).

• Gracely Pain Scale: A 13-point scale that quantifies pain intensity to ensure patient comfort.

• NK cell count: Measures immune defense (42).

Together, these outcome indicators form a multidimensional scoring system that not only monitors the disease status and treatment response of HIV-infected individuals but also provides in-depth insights into the quality of life and overall well-being of patients.

### 2.3 Exclusion criteria

Exclusion criteria were as follows: conditions unrelated to HIV/AIDS and its associated symptoms: the treatment group receives non-traditional CM therapies, while the control group is administered other therapeutic methods; the absence of relevant outcome measures: reviews, systematic evaluations, theoretical discussions, case reports, animal studies, uncontrolled observational research, and low-quality observational studies; and incomplete datasets and duplicated publications or research findings.

### 2.4 Search strategy

A systematic search was conducted across four databases: PubMed, Embase, Web of Science, and the Cochrane Library were searched, focusing on randomized controlled trials and observational studies concerning the treatment of HIV/AIDS with traditional Chinese medicine. The search was conducted from the inception of the databases to 27 August 2024. Furthermore, the reference lists of the identified studies were reviewed to ensure that no relevant studies had been overlooked. The English search terms utilized included “Medicine, Chinese Traditional,” “Drugs, Chinese Herbal,” “Acupuncture,” “Pharmacopuncture,” “Electroacupuncture,” “Moxibustion,” “Acupuncture Points,” “Meridians,” “Massage,” “Cupping Therapy,” “Heat Therapy,” “Fumigation,” “Preprint,” “Citation Index,” “Randomized Controlled Trial,” “Case Control Study,” “Cohort Analysis,” and “Cohort Study.” The complete search strategy is detailed in Supplementary Appendix S1.

### 2.5 Study selection and data extraction

Two reviewers used EndNote X9.1 software to screen the included literature independently. Duplicate records were initially removed, and the titles and abstracts of each document were then reviewed based on established inclusion and exclusion criteria. Full-text reviews were conducted for the selected literature that met the criteria to determine its inclusion in the analysis. Data were collected in Excel, including the first author, publication year, sample size (for both the intervention and control groups), age, intervention measures (for the intervention and control groups), duration of treatment, and outcome indicators.

### 2.6 Bias risk assessment

Risk of bias in randomized controlled trials was assessed using the Cochrane Risk of Bias tool, which meticulously analyzes seven key areas (sequence generation, allocation concealment, blinding of participants and personnel, blinding of outcome assessment, completeness of outcome data, selective reporting, and other biases). For observational studies, we employed the Newcastle-Ottawa Scale to assess the risk of bias. Each criterion was strictly reviewed, and the results of the randomized controlled trials were visually presented using ReviewManager 5.4.1 software. The results of observational studies were obviously displayed through a three-line table format. The two reviewers exchanged and thoroughly compared their respective assessment outcomes, resolving any discrepancies through collective discussions within the research team.

### 2.7 Statistical methods

Utilizing R version 4.2.2 software to call the BUGSnet 1.1.0 package to perform Bayesian Network Meta-Analysis, general linear models are simulated through JAGS 4.3.1 for sampling (43). The effect size for continuous variables is estimated using the mean difference (MD), and for binary variables, the odds ratio (OR) is used to estimate the effect size. Results are presented as the effect size with its 95% Bayesian credible interval (95% CrI). Model selection is based on the leverage plot and the deviance information criteria (DIC). A model is considered to have a better fit when the data points on the leverage plot are within the purple dashed line (*x* squared + *y* = 3) and when the DIC is relatively lower (a difference in DIC <3 between models is considered non-significant, and a fixed-effect model is used; when the difference in DIC is ≥3, the model with the lower DIC is selected) (44). The cumulative ranking probability plot is evaluated using the nma.rank() function to calculate the surface under the cumulative ranking (SUCRA) to assess the ranking of interventions. The nma.league() function is used to obtain the ranking table, and the ggplot2 package is utilized to output the results as a SUCRA plot and a ranking table heatmap. The analysis results are presented as a forest plot using the nma.forest() function. The presence of publication bias is tested using the comparison-adjusted funnel plot through Stata 17.0.

## 3 Results

### 3.1 Study selection and study characteristics

**Figure 1:**
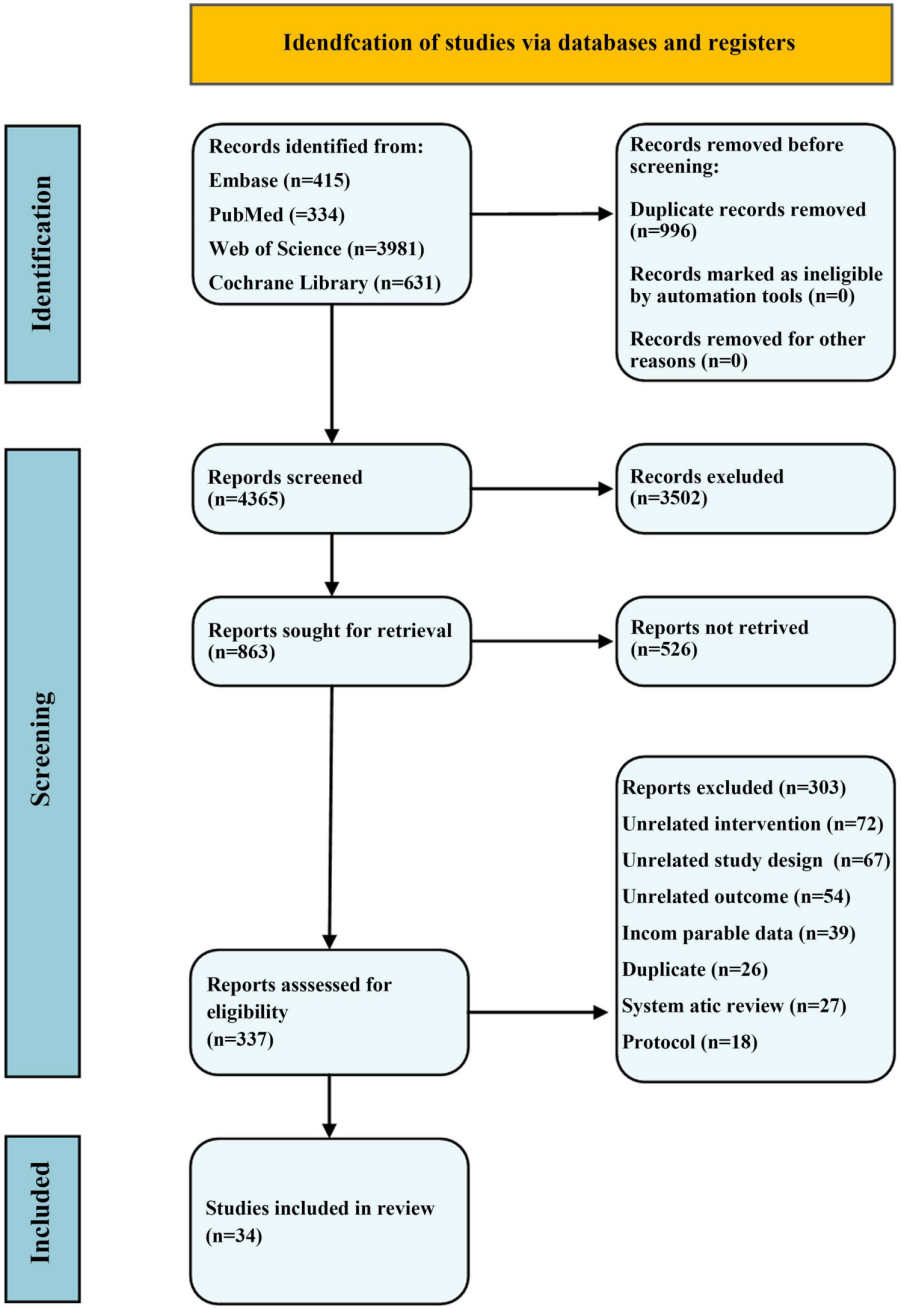
Flow diagram of the selection process.

An initial review of the titles and abstracts identified 337 articles. After a comprehensive assessment of the full texts, 303 were excluded due to the presence of irrelevant interventions, diagnostic criteria, or non-research literature. Ultimately, 34 studies were selected for analysis (Figure 1).

The aggregate sample size encompassed a total of 8,933 individuals, with the treatment cohort comprising 4,542 patients and the control cohort consisting of 4,391 individuals. The investigation encompassed a spectrum of 16 distinct interventions, which included Chinese herbal formulae, single herbs, Chinese plants extracts, acupuncture, ART, no treatment, interventions based on modern medical practices, placebo administrations, absence of treatment, combinatorial therapies involving Chinese herbal formulae with ART, Chinese herbal formulae with Western medical therapeutics (WMT), placebo with ART, placebo with Western medical therapeutics, placebo with Western medical placebos, and acupuncture with Western medical placebos. Tables 1 and 2 delineate the specific characteristics of the studies incorporated within this comprehensive analysis.

**Table 1:** Characteristics of included studies (1).

No.	Study	Intervention				Number of patients	Intervention period	Relevant outcomes
		T			C			
1	Cao Wei (45)	CPE			PL	149 (46/51/52)	48 weeks	①②
2	Zhuang Tao (46)	CHF + ART			PL + ART	47 (24/23)	24 weeks	①⑦
3	Aisha Gambo (47)	SH			PL	177 (89/88)	6 months	①③⑥
4	Dongli Wang (48)	CHF + ART			ART	739 (233/506)	14years	①
5	Oumarou Goni Hamadama (49)	CHF	CHF + ART		ART	297 (97/100/100)	6 months	①②
6	SU Qi-jian (50)	CHF + ART			ART	300 (100/100/100)	1 year	①②③⑧
7	Jin Sun (20)	CHF	CHF + ART		ART	142 (52/59/31)	4 years	①②
8	XU Qian-lei (51)	CHF + ART			ART	275 (95/180)	1 year	①④
9	Young-Keol Cho (52)	SH			NT	252 (162/90)	86 months	①④
10	Reychler Gregory (53)	MT			PL	29 (15/14)	4 weeks	⑤
11	Barbara Swanson (54)	AC			PL	25 (12/13)	8 weeks	①③
12	Yantao Jin (55)	CHF + ART			ART	3,229 (1,442/1,787)	96 months	④
13	Ma Xiu-xia (56)	CHF + WMT			WMT	130 (85/45)	4 weeks	⑧
14	Jian Wang (57)	CHF + ART			CHF	120 (42/78)	84 months	①⑧
15	Xu Wen-fang (58)	CHF + ART			ART	45 (23/22)	24 weeks	①⑧
16	Liu Zhen-wei (59)	AC			WMT	60 (30/30)	2 weeks	⑧
17	Cen Yu-wen (60)	CHF			WMT	160 (110/50)	4 weeks	⑧
18	Liu Zhen (61)	CHF			PL	223 (116/117)	6 months	①②⑧
19	Wu Xin-fang (62)	CHF + ART			PL + ART	187 (98/89)	6 months	①⑧
20	Wang Jie (63)	CHF + ART			PL + ART	233 (116/117)	6 months	①
21	Mangaiarkarasi Asokan (64)	CHF			ART	38 (13/25)	24 months	①
22	Joyce K. Anastasi (65)	AC			PL	50 (25/25)	6 weeks	⑦
23	Xu Li-ran (66)	CHF			PL	1,155 (769/386)	18 months	④
24	Jiang Shi-qing (67)	CHF + ART			PL + ART	115 (57/58)	6 months	⑧
25	Lu Zi-yun (68)	CHF			PL	71 (41/30)	22 months	①②③⑥
26	S.C. Wright (69)	SH			WMT	52 (18/17/17)	11 days	⑧
27	Jiang Feng (70)	CHF			WMT	80 (40/40)	2 weeks	⑧
28	M.V. Kalikar (71)	CPE			PL	60 (31/29)	6 months	①
29	Wang Jian (72)	CHF			PL	63 (30/33)	6 months	①②
30	Gail Shor-Posner (73)	MT			PL	24 (10/14)	12 weeks	①②
31	Rainer Weber (74)	CHF			PL	42 (20/22)	6 months	①③
32	Judith C. Shlay (75)	AC	AC + WMT	AC + WMPL	PL PL + WMT PL + WMPL	239 (58/32/31/56/33/29)	14 weeks	⑧
33	J. Durant (76)	CPE			PL	145 (49/48/48)	37 weeks	①③
34	Jeffrey H. Burack (77)	CHF			PL	29(15/14)	12 weeks	①⑥⑧

AC, acupuncture; ART, antiretroviral therapy; C, control group; CHF, Chinese herbal formulae; CPE, Chinese plant extracts; MT, massage; NT, no treatment; PL, placebo; SH, single herb; T, treatment group; WMPL, Western medical placebo; WMT, Western medical therapeutics.

① CD4 cell count, ② CD8 cell count, ③ HIV viral load, ④ Quality of life (QoL) scale, ⑤ Body weight (including body mass index—BMI), ⑥ Pain scales (Global Pain Scale—GPS and Self-Reported Pain Numeric Scale—SPNS), ⑦ Survivor count, ⑧ Overall efficacy (treatment effectiveness).

**Table 2:** Characteristics of included studies (2).

Study	Year	Study period	Age (years)/mean (SD)		
			T		C
Cao Wei (45)	2023	2019.08–2022.07	41.92 (8.14)	41.43 (7.77)	44.17 (8.41)
Zhuang Tao (46)	2022	2019.07–2019.11	47 (10.8)		42.8 (11.2)
Aisha Gambo (47)	2021	2017.12–2018.11	NA		
Dongli Wang (48)	2021	2004.04–2019.04	40.8 (8.39)		40.0 (7.96)
Oumarou Goni Hamadama (49)	2021	NA	NA		
Su Qijian (50)	2020	2015.02–2016.06	48.5 (14.3)	45.2 (16.1)	48.3 (15.4)
Jin Sun (20)	2019	NA	47.41 (14.74)	47.93 (13.00)	45.12 (16.13)
Xu Qianlei (51)	2018	2013.09–2014.09	50.1 (6.6)		48.8 (6.2)
Young-Keol Cho (52)	2017	NA	30.3 (9.6)		32.5 (9.2)
Reychler Gregory (53)	2017	2015.06–2015.12	45.5 (10.9)		47.1 (16.6)
Barbara Swanson (54)	2015	2011.07–2012.10	47.69 (4.8)		47.33 (9.1)
Yantao Jin (55)	2014	2004.10–2012.10	NA		
Ma Xiuxia (56)	2014	2009.04–2011.03	49.18 (8.72)		49.13 (7.55)
Jian Wang (57)	2014	NA	NA		
Xu Wenfang (58)	2014	2010.01–2012.12	41.4 (13.4)		39.1 (12.8)
Liu Zhenwei (59)	2013	2010–2011	44 (21)		46 (18)
Cen Yuwen (60)	2013	2009.10–2011.03	44.43 (10.53)		44.71 (9.95)
Liu Zhen (61)	2013	2009.09–2010.09	42.08 (10.41)		42.3 (11.98)
Wu Xinfang (62)	2013	2009.09–2010.06	37.38 (10.97)		39.15 (10.12)
Wang Jie (63)	2013	2009.09–2010.05	42.08 (10.41)		42.3 (11.98)
Mangaiarkarasi Asokan (64)	2013	2006.06–2009.08	34.46 (4.84)		36.58 (5.6)
Joyce K. Anastasi (65)	2013	NA	47.8 (7.2)		47.76 (7.5)
Xu Liran (66)	2013	2009.06–2010.12	NA		
Jiang Shiqing (67)	2011	NA	42.4 (9)		43.6 (8.25)
Lu Ziyun (68)	2011	NA	39.7 (10.3)		37.6 (7.8)
S.C. Wright (69)	2009	NA	NA		
Jiang Feng (70)	2009	NA	44.6 (7)		43.3 (7.5)
M.V. Kalikar (71)	2008	NA	NA		
Wang Jian (72)	2006	2002.06–2003.07	NA		
Gail Shor-Posner (73)	2004	2003.06–2003.10	NA		
Rainer Weber (74)	1999	1996.02–1996.09	35.44 (6.46)		36.88 (6.69)
Judith C. Shlay (75)	1998	1993.05–1995.03	NA		
J. Durant (76)	1998	1995.01–1995.10	35 (11)		34 (8)
Jeffrey H. Burack (77)	1996	NA	34.5 (2)		37.7 (2.4)

C, control group; SD, standard deviation; T, treatment group.

Gail Shor-Posner (73) conducted a pediatric study; however, no baseline age characteristics of the participants were provided. In contrast, all other studies included in our analysis exclusively enrolled adult patients (≥18 years).

### 3.2 Bias risk assessment

Of the 34 selected studies, 27 were randomized controlled trials and seven were observational studies, all of which were cohort studies.

#### 3.2.1 Randomized controlled trials

**Figure 2:**
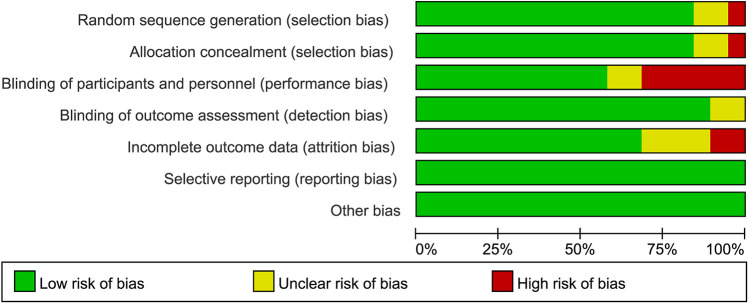
Risk of bias graph.

**Figure 3:**
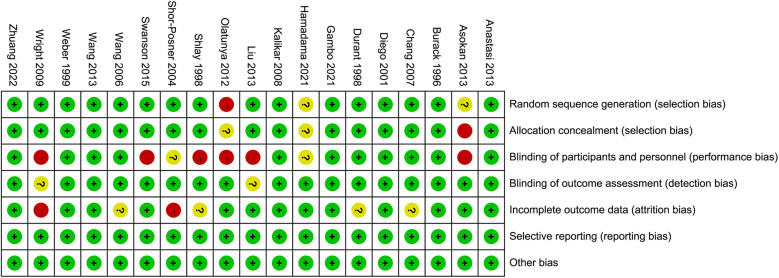
Risk of bias summary. Green: low risk of bias; Yellow: some concerns; Red: high risk of bias.

The risk of bias graph and risk of bias summary for the included randomized controlled trials can be found in Figures 2 and 3.

Allocation: a total of 17 studies (45, 47, 53, 54, 56, 59–65, 67, 70, 74–77) provided adequate information on their random sequence generation, thereby considered at low risk of bias, primarily using computer programs or random number tables to generate sequences. Eight studies (46, 58, 66, 68, 69, 71–73) were described as being randomized but gave insufficient information as to how a random sequence was generated, and so were deemed at unclear risk of bias. In addition, two studies (49, 64) were characterized as non-randomized, thus being perceived as having a higher risk of bias. In total, 11 studies (45, 47, 54, 60–63, 65, 70, 74, 75) gave adequate information on their method of allocation concealment to be deemed at low risk of bias, utilizing either third party central allocation or sealed opaque envelopes. In total, 14 studies (46, 53, 56, 58, 59, 66–69, 71–73, 76, 77) did not provide information on how allocation concealment was achieved, and so were deemed at unclear risk of bias. In addition, two studies (49, 64) were deemed to be at a higher risk of bias due to the absence of randomization, which rendered allocation concealment unfeasible.

Blinding: a total of 14 studies (45–47, 61–63, 67, 68, 71, 72, 74, 76, 77) described in sufficient detail their method of blinding participants and trial personnel to be deemed at low risk of performance bias. Four studies (53, 54, 65, 75) also indicated that participants and personnel were blinded, but failed to specify how this was achieved, thus presenting an unclear risk of bias. One study (66) stated that participants and personnel were blinded, but did not state how this was achieved, and so were deemed at unclear risk of bias. Eight studies (49, 56, 58–60, 69, 70, 73) either made no mention of blinding of participants and personnel or openly stated that participants were not blinded to interventional arms; therefore, they were deemed at high risk for performance bias. In total, 17 studies (45–47, 53, 61–65, 67, 68, 71, 72, 74–77) described in sufficient detail their method of blinding outcome assessors to be deemed at low risk of detection bias. Two studies (54, 66) were described as blinded but did not describe how outcome assessors were blinded to participant allocation, and so were deemed at unclear risk of bias. Eight studies (49, 56, 58–60, 69, 70, 73) either made no mention of blinding of outcome assessors or openly stated that assessors were not blinded to interventional arms; therefore, they were deemed at high risk for detection bias.

Incomplete outcome data: of the studies, 24 (45, 47, 49, 53, 54, 56, 58, 59, 61–71, 73–77) adequately reported their trial flow, with reasons given for withdrawals and balanced withdrawals across interventional arms, and were deemed at low risk for attrition bias. Three studies (46, 60, 72) did not provide sufficient information for attrition through the study process to be assessed and were deemed at unclear risk of bias.

Selective reporting: nine studies (45–47, 53, 58, 61, 63, 66, 69) reported their outcomes appropriately per their trial registrations. The other 18 studies (49, 54, 56, 59, 60, 62, 64, 65, 67, 68, 70–77) either did not have trial registrations or did not fully appropriately report outcome data.

Other potential sources of bias: a total of 25 (45–47, 49, 53, 56, 58–72, 74–77) studies were at low risk of bias for other bias as there were no baseline imbalances per group, or other imbalances affecting outcome data. Only two studies—Gail Shor-Posner 2004 (73) and Barbara Swanson 2015 (54)—were rated as having an unclear risk of bias due to partial baseline imbalance in outcome measures across groups.

#### 3.2.2 Observational studies

The risk of bias assessment for the included observational studies is depicted in Table 3.

**Table 3:** Table risk of bias for included studies—observational studies (cohort studies).

Study	Selection of cohorts				Comparability	Outcome			Total
	Representativeness of the exposed cohort	Selection of the non-exposed cohort	Ascertainment of exposure	Demonstration that outcome of interest was not present at start of study	Comparability of cohorts on the basis of the design or analysis	Assessment of outcome	Was follow-up long enough for outcomes to occur	Adequacy of follow up of cohorts	Overall Quality
Dongli Wang (48)	★	★	★	★		★	★		Moderate
Su Qijian (50)		★		★			★	★	Moderate
Jin Sun (20)	★	★	★	★		★	★		Moderate
Xu Qianlei (51)		★	★	★		★	★		Moderate
Young-Keol Cho (52)	★	★		★	★		★	★	Moderate
Yantao Jin (55)	★	★	★	★		★	★	★	High
Jian Wang (57)	★	★		★			★		Moderate

★Studies with scores of 7–9 were deemed high-quality, those with scores of 4–6 were considered moderate-quality, and those with scores below 4 were classified as low-quality.

##### 3.2.2.1 Selection (study object selection)

Adequacy of case definition: Five studies (20, 48, 52, 55, 57) employed independent ascertainment methods or personnel, earning a one-star rating. Two additional studies (50, 51) lacked clear or appropriate descriptions of case selection, receiving a zero-star rating.

Representativeness of the cases: Seven studies (20, 48, 50–52, 55, 57) demonstrated the continuity or representativeness of cases, aligning with the characteristics of the general population, thus receiving a one-star rating.

Selection of controls: Four studies (20, 48, 51, 55) utilized control groups from the community (entire population), securing a one-star rating; whereas three other studies (50, 52, 57) relied on hospital-based controls or lacked descriptions, resulting in a zero-star rating.

Definition of controls: Seven studies (20, 48, 50–52, 55, 57) successfully demonstrated that the outcome of interest was absent at the study's inception, meriting a one-star rating.

##### 3.2.2.2 Comparability

Comparability of cases and controls based on design or analysis: Only one study (52) considered the comparability of cases and controls in its design and statistical analysis, controlling for the most significant confounders, and thus received a one-star rating; the remaining six studies (20, 48, 50, 51, 55, 57) did not account for or control confounding factors, receiving a zero-star rating.

##### 3.2.2.3 Outcome (outcome measurement)

Adequacy of outcome assessment: Four studies (20, 48, 51, 55) provided clear documentation from medical records (e.g., surgical logs) or conducted structured interviews blinded to the individual's case or control status, earning a one-star rating; three other studies (50, 52, 57) failed to implement blinded interviews, relied solely on written self-reports or complaints, or lacked descriptions, receiving a zero-star rating.

Same method of ascertainment for cases and controls: Seven studies (20, 48, 50–52, 55, 57) employed identical methods to determine the exposure factors for both cases and controls, receiving a one-star rating.

Non-response rate: Three studies (50, 52, 55) had equivalent non-response rates between cases and controls or described the circumstances of non-respondents, earning a one-star rating; four other studies (20, 48, 51, 57) showed differing non-response rates without descriptions, resulting in a zero-star rating.

Overall risk of bias: Based on NOS scoring, one study (55) achieved a total of 7 points, indicating a low risk of bias; three studies (20, 48, 52) received 6 points, one study (51) received 5 points, and two studies (50, 57) received 4 points, all categorizing them as studies with a moderate risk of bias.

### 3.3 Network meta-analysis of results

**Figure 4:**
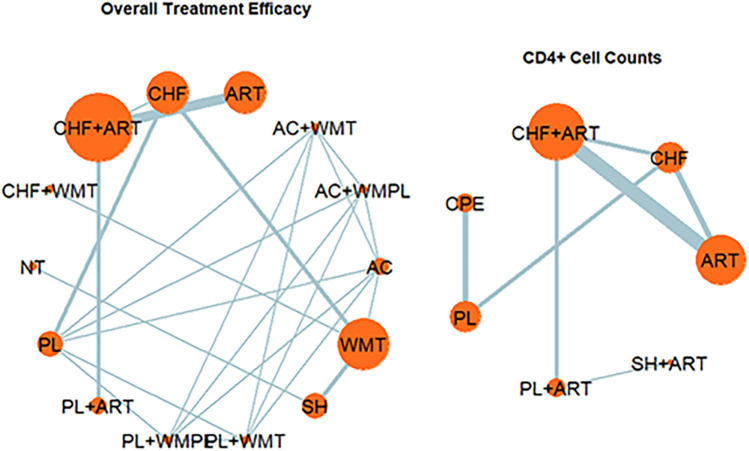
Network plot of treatment comparisons in the network meta-analysis.

**Figure 5:**
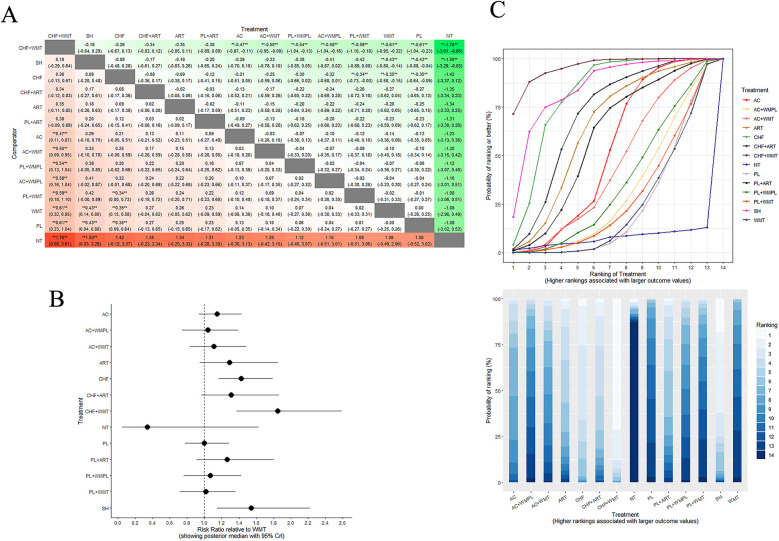
Comparison of overall treatment efficacy between WMT and other therapeutics. **(A)** League table: this chart presents the relative effectiveness of all intervention pairs along with their 95% CrI, enabling comparison between any two treatments. The symbol (**) in the figure indicates that there were significant differences between the interventions (*p* < 0.05). **(B)** Forest plot: this figure illustrates the effect of each intervention compared to WMT. The dashed line indicates the line of no effect, and the horizontal lines represent confidence intervals. **(C)** SCURA curve and Rankogram plot: presented as a line graph and bar chart, these plots show the ranking probabilities for each intervention. WMT, Western medical therapeutics.

**Figure 6:**
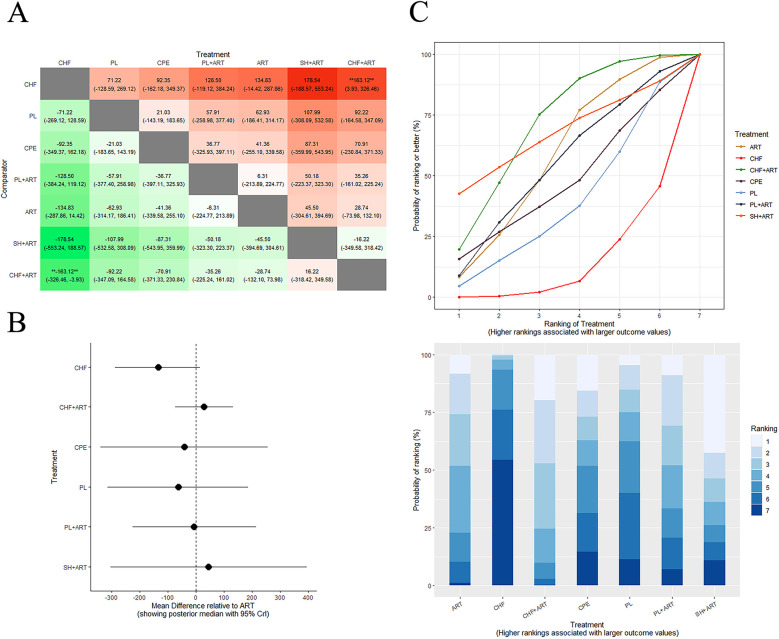
Comparison of CD4^+^ cell counts between ART and other therapeutics. League table: this chart presents the relative effectiveness of all intervention pairs along with their 95% CrI, enabling comparison between any two treatments. The symbol (**) in the figure indicates that there were significant differences between the interventions (*p* < 0.05). **(B)** Forest plot: this figure illustrates the effect of each intervention compared to ART. The dashed line indicates the line of no effect, and the horizontal lines represent confidence intervals. **(C)** SCURA curve & Rankogram plot: presented as a line graph and bar chart, these plots show the ranking probabilities for each intervention. ART, antiretroviral therapy.

Figure 4 presents the NMA chart illustrating the comparative impacts of various therapeutic interventions on CD4^+^ cell counts and overall treatment efficacy, which includes survival and improvement rates. The lines connecting the nodes in the chart denote direct comparisons between pairs of interventions. The size of each node is indicative of the sample size encompassed within each intervention, while the thickness of the lines represents the number of studies incorporated in the comparison between the two interventions. Figure 5 provides an exhaustive matrix detailing the outcomes. Figure 6 displays the Surface Under the Cumulative Ranking (SUCRA) curve results, which rank all treatment plans under scrutiny. A higher SUCRA score for CD4^+^ cell counts and overall treatment efficacy signifies that the intervention is more effective in enhancing the immunological restoration and quality of life for HIV/AIDS patients. The meta-analysis was precluded from the study by Gail Shor-Posner (73) due to the absence of comparable pediatric studies; it was therefore presented only as contextual evidence.

#### 3.3.1 Consistency check

In the studies included in this analysis, while most treatment modalities were compared, there remains a gap in direct comparisons between certain interventions, such as single herbs vs. Chinese herbal formulae plus ART, acupuncture vs. Chinese herbal formulae, and acupuncture vs. single herbs. This gap poses an obstacle to the formation of a closed loop. When evaluating overall treatment efficacy, the deviance information criterion (DIC) values for both the random-effects and fixed-effects models were close, with the fixed-effects model having a slightly lower DIC value than the random-effects model. However, given the substantial number of articles included in this study, the random-effects model was ultimately selected (78) (Supplementary Figure S1). For the CD4 count, the random-effects model was superior to the fixed-effects model, with lower DIC values and fewer outliers in the leverage plot (Supplementary Figure S2).

In the consistency model, the DIC values were smaller, and the leverage plots appeared clearer (Supplementary Figure S3). With the exception of a few outliers, the majority of data points were located on or near the line (*y* = *x*), indicating a high degree of consistency between the two models (Supplementary Figure S4). Therefore, it is recommended to utilize the concise consistency model for analysis.

#### 3.3.2 Overall treatment efficacy

In the cohort of 34 articles reviewed, three reported on survival rates and 13 on improvement rates. As depicted in Figure 5A, compared to Western medicine treatment, the combination of Chinese herbal formulas with Western medical treatment (MD = 5.45, 95% CrI: 1.06–37.08), single herbs (MD = 4.46, 95% CrI: 1.03–26.91), and Chinese herbal formulas (MD = 4.13, 95% CrI: 0.89–29.06) demonstrated the most significant therapeutic effects, survival rates, and improvements in quality of life. These findings underscore the enhanced efficacy of integrating CM with modern medicine approaches in augmenting immunological restoration and overall well-being for patients with HIV/AIDS. According to the SUCRA curve and Rankogram plot (Figure 2C), the effectiveness in overall treatment is as follows: “CHF + WMT” > “SH” > “CHF” > “CHF + ART” > “ART” > “PL  +  ART” > “AC” > “AC  +  WMT” > “PL  + WMPL” > “AC + WMPL” > “PL + WMT” > WMT” > “PL” > “NT”; the three most effective measure for overall treatment is “CHF + WMT,” “SH,” and “CHF,” while the least effective is “NT.”

#### 3.3.3 CD4^+^ cell counts

Of the 23 included articles, 14 reported on CD4^+^ cell counts. As depicted in Figure 6A, compared with ART, the combination of Chinese herbal formulae plus ART (MD = 163, 95% CrI: 3.93–326.46) and single herbs plus ART (MD = 178.54, 95% CrI: −188.57–553.24) showed the most significant effects on restoring CD4^+^ cell counts and improving immunological restoration in HIV patients. According to the SUCRA curve and Rankogram (Figure 6C), the effectiveness in restoring CD4^+^ cell counts in HIV patients is ordered as follows: “CHF + ART” > “SH + ART” > “ART” > “PL + ART” > “CPE” > “PL” > “CHF.” The two most effective measures in restoring CD4^+^ cell counts and improving immunological restoration in HIV patients are “CHF + ART” and “SH + ART,” while the least effective measure is “CHF.”

## 4 Discussion

In this systematic review incorporating treatment-level and immune reconstitution-level NMA of 27 randomized controlled trials and seven cohort studies (involving >8,900 participants), we observed consistent directional evidence that CM combined with ART or Western drugs may meaningfully enhance immune reconstitution, reduce HIV-related complications and ART-associated adverse effects, and potentially improve health-related quality of life and functional recovery in patients with suboptimal immune restoration. Although the overall quality of included trials and cohort studies was acceptable, the limited number of studies and participants, coupled with risk of bias and imprecision in some reports, reduced the certainty of our effect estimates. Consequently, more multicenter trials with low risk of bias are required to confirm the benefits and generalizability of CM combined with ART and Western drugs for HIV treatment. Patients, clinicians, and health system leaders may concurrently consider integrating evidence-informed CM interventions into HIV management protocols.

### 4.1 The paradox of monotherapy vs. Synergistic Integration

Our analysis revealed a critical dichotomy: Chinese herbal formulae combined with ART demonstrated superior efficacy in CD4^+^ immune reconstitution and overall survival, whereas Chinese herbal formulae monotherapy yielded suboptimal immunological outcomes. This paradox underscores CM's fundamental role—not as a substitute for ART, but as an advanced immunoadjuvant therapy. The limited efficacy of isolated CM interventions stems from its unique pharmacodynamic characteristics: Unlike ART's direct viral suppression, botanicals like Astragalus membranaceus (79) or Salvia miltiorrhiza (80) modulate immune pathways through cytokine regulation (e.g., IL-2/IFN-γ downregulation) and hematopoietic progenitor activation—mechanisms easily overwhelmed by uncontrolled viral replication. Crucially, CM's immune-reparative potential is unlocked only when antiviral therapy achieves virological suppression, which explains the CD4^+^ advantage of 163 cells/μL (95% CrI: 3.93–326.46) for CHF + ART regimens. This synergy proves particularly transformative for 20%–30% of ART-adherent individuals.

### 4.2 Immune reconstitution: TCM's unmatched niche

Despite ART's benefits, 20% of long-term recipients experience incomplete Immunological non-responder (INR) with suboptimal CD4^+^ T-cell recovery (81). CM addresses immune dysregulation through multidimensional mechanisms: Wenshen Jianpi formula promotes CD4^+^ T-cell counts and reverses HIV-induced NK-cell imbalance (46); bioactive compounds (e.g., LLDT-8 from *Tripterygium wilfordii*) reduce CD8^+^ T-cell activation markers, suppress IFN-α/γ signaling, and expand Treg populations (82). Concurrently, *Angelica sinensis*'s ethyl acetate fraction inhibits macrophage proinflammatory mediators, its volatile oils exert antioxidant effects, and its polysaccharides suppress COX-1 activity (83); *A. dahurica* inhibits histamine release (84) and elastase activity/expression (85) while blocking LPS-induced TNF-α/NO/PGE₂ production (86); *Scutellaria baicalensis* flavonoids (baicalin and baicalein) suppress inflammation (87); *Codonopsis pilosula* polysaccharides activate Keap1-Nrf2 to elevate SOD/GSH-Px activity (88, 89) and inhibit proinflammatory activities (90); *Astragalus membranaceus* flavonoids reduce ROS release (91), and its saponins/polysaccharides attenuate hepatic fibrosis via TGF-β/Smad inhibition (92); the pachymic acid in *Poria cocos* mitigate renal injury by suppressing ferroptosis (93); and *Forsythia suspensa* glycosides modulate intestinal flora via TGF-β1/Smad3 inhibition to alleviate hepatic fibrosis (94). This integrative profile—simultaneously targeting inflammation, oxidation, fibrosis, and immune regulation—constitutes CM's irreplicable value in the ART era (95).

These multi-level effects are fundamentally mediated by precise molecular mechanisms. CM adjunct therapy modulates critical HIV-associated pathways through specific bioactive compounds. Key mechanisms include suppression of proinflammatory cytokines (e.g., IL-6, TNF-α) via NF-κB pathway inhibition (96), amelioration of oxidative stress through Nrf2-mediated antioxidant activation, and metabolic regulation (97–99). These effects are materially mediated by constituents such as astragalosides (immunomodulation via TLR4/MyD88), salvianolic acids (anti-fibrotic via TGF-β/Smad), and baicalin (anti-inflammatory via COX-2/iNOS suppression), collectively enabling multi-target immunometabolic regulation complementary to ART (100–102).

### 4.3 Mastery of comorbidities and ART toxicities

CM demonstrates unparalleled efficacy in managing ART-induced comorbidities. Facing ART-induced peripheral neuropathy, acupuncture reduced Gracely pain scores by 0.7 points vs. sham acupuncture at 24 weeks, outperforming gabapentin in drug-resistant cases (75). In addition, Moringa leaves correct malnutrition through amino acid supplementation (103), counters oxidative stress via isothiocyanates (104), and alleviates enteric neuropathy through acetylcholinesterase inhibition (105). This multi-target, multi-pathway action mode constitutes a critical factor in CM's efficacy—unattainable by single-molecule drugs or conventional Western pharmacotherapy alone.

### 4.4 Methodological imperatives and future vectors

Although CM interventions can effectively increase CD4^+^ T-cell counts and represent a promising approach for treating incomplete INR, their mechanisms in immune recovery remain to be elucidated (106). Translating CM's therapeutic potential into evidence-based practice requires urgent resolution of three fundamental gaps: unquantified pharmacokinetic interactions between herbal compounds and antiretroviral drugs necessitate population pharmacokinetic (PK)/pharmacodynamic (PD) modeling to prevent compromised clinical efficacy; standardization crises arising from batch-to-batch variability of bioactive components demand chemical fingerprinting and bioactive equivalence metrics to ensure therapeutic consistency; and mechanistic opacity surrounding herbal actions across different HIV disease stages calls for structural biology approaches to deconvolve molecular targets. Future research must prioritize rational combination strategies leveraging phytochemical synergies, multidimensional endpoints (including immune profiling and reservoir quantification), and bioavailability-enhanced formulations to overcome pharmacokinetic barriers—all implemented through adaptive trial designs focusing on incomplete immune responders, with personalization of herb-ART synergies guided by host biomarkers.

### 4.5 Comprehensive considerations on intervention heterogeneity

The interventions included in this study encompass a range of complementary and alternative medicine modalities from CM, as well as various modern medical approaches. While this diversity introduces complexity, it accurately reflects real-world CM clinical practice. From a theoretical perspective, these therapies are all derived from a unified CM theoretical framework. For instance, for the HIV-related pattern of “Yin deficiency of the Liver and Kidney,” an herbal formula may act internally to nourish these organs, while acupuncture might stimulate specific meridian points [e.g., Taixi (KI3), Sanyinjiao (SP6)] to activate channel Qi—both exemplifying the principle of “internal and external combination therapy.”

Although their pathways differ—herbal medicine primarily via multi-target chemical regulation, acupuncture through physical stimulation of neural and bioelectrical signals, and tuina via mechanical adjustment of musculoskeletal structures—they may ultimately influence HIV progression by modulating shared core pathophysiological processes. From a modern medical standpoint, these shared processes may include (1) immune regulation, such as increasing CD4^+^ T-cell counts, modulating the CD4^+^/CD8^+^ ratio, and enhancing NK cell activity to promote immune reconstruction (107); (2) control of chronic immune activation and inflammation, by downregulating key proinflammatory cytokines (e.g., TNF-α, IL-6) to mitigate persistent immune damage (108); (3) amelioration of metabolic disorders and oxidative stress, thereby alleviating ART-related side effects such as hepatotoxicity and dyslipidemia (109).

### 4.6 Strengths and limitations

This review was conducted in adherence to the rigorous standards of systematic reviews and network meta-analysis. A key strength was the *a priori* development and registration of our protocol in consultation with clinicians/researchers. Our analysis encompassed a broad spectrum of CM therapeutics and modern medicine treatments, and provided insights into both monotherapy and combination therapies. By integrating evidence from 27 trials and seven cohort studies involving over 8,900 participants, this work offers a comprehensive evidence map reflective of the “holistic concept” often applied in clinical CM practice for HIV management.

However, the findings must be interpreted in the context of several important limitations. The certainty of the evidence was limited by the relatively small number of available studies for specific interventions. A notable risk of bias was present, primarily due to challenges in blinding within CM trials. Furthermore, substantial heterogeneity existed among the included interventions, which vary in their modes of action (e.g., chemical, physical). The limited number of studies within each specific intervention category precluded more detailed subgroup analyses. Imprecision was another major constraint, stemming from sparse data, particularly for herbal formulas combined with Western drugs, acupuncture combined with Western drugs, single herb interventions, and outcomes pertaining to placebos. This sparseness of data constrained the robustness of our efficacy estimations. Finally, a critical limitation for clinical safety is that most included studies did not report detailed PK/PD interaction data between CM components and antiretroviral drugs, leaving a significant evidence gap on potential herb–drug interactions. In addition, this study was specifically framed within the theoretical system of Chinese Medicine rather than encompassing global traditional medicine practices. Future research should therefore explore comparative effectiveness across different traditional medicine systems.

### 4.7 Future perspectives

To translate the promising potential of CM in HIV care into definitive clinical guidelines, future research should follow a coherent and translational pathway. The priorities for future investigation are multi-faceted.

First, to address the critical evidence gap on safety, rigorous herb–drug interaction studies are essential. Future research on the development of standardized CM preparations or active ingredient extracts should prioritize the following: beginning with *in vitro* models (e.g., liver microsomes, high-throughput screening) to evaluate the effects of common herbs and their active compounds on key ART metabolic pathways (e.g., CYP450 enzymes, drug transporters) (110–112); followed by preclinical PK/PD studies in animal models to establish preliminary safety and dosing guidance (113); and ultimately, incorporating PK sub-studies within clinical trials to directly assess interaction potential between standardized CM preparations and specific ART regimens.

Second, high-quality mechanistic studies should be prioritized. Employing modern pharmacological approaches such as network pharmacology and molecular docking can help identify the key targets and signaling pathways of core herbal formulae, providing a scientific rationale for their observed efficacy.

Subsequently, the field requires well-designed, large-scale, multicenter, randomized double-blind placebo-controlled trials. These trials must target individual intervention modalities and utilize standardized interventions (e.g., fixed-formula concentrated granules, uniform acupuncture protocols), objective biological endpoints (e.g., CD4/CD8 ratio, inflammatory cytokine profiles), and validated patient-reported outcomes to generate high-quality efficacy evidence and allow for more granular subgroup analyses.

**Figure 7:**
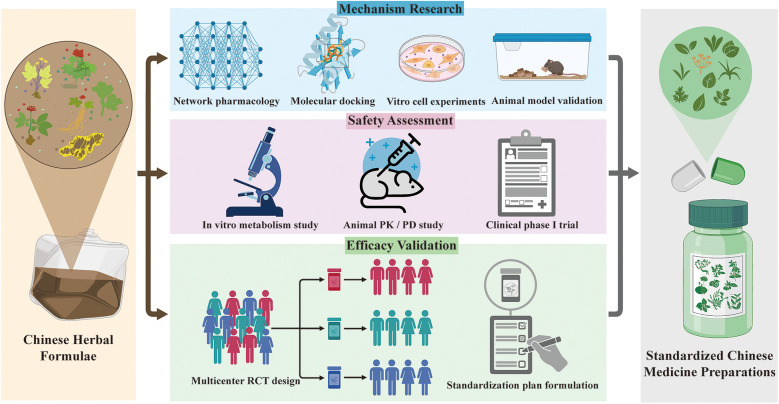
Structured research roadmap—progressing from “mechanism” to “safety” to “efficacy.” Classical Chinese herbal formulae can be refined through mechanistic studies—such as network pharmacology, molecular docking, *in vitro* experiments, and animal studies—to streamline their composition. Subsequent safety evaluation involves *in vitro* metabolism studies, animal PK/PD investigations, and Phase I clinical trials. This is followed by multicenter randomized controlled trials to establish standardized protocols. Ultimately, this process drives the transformation of herbal formulations into standardized CM preparations, thereby providing higher-level evidence for safer and more effective integrative medicine guidelines.

Through this structured research roadmap (Figure 7)—progressing from “mechanism” to “safety” to “efficacy”—the definitive role of CM as an adjunctive therapy in comprehensive HIV care can be established. This effort will ultimately contribute to offering people living with HIV more diversified and personalized therapeutic options to enhance their long-term quality of life.

## 5 Conclusions

In this systematic review with treatment-level network meta-analysis, we found evidence suggesting CM modalities—particularly herbal formulae combined with ART or conventional pharmacotherapy—may effectively restore immune reconstitution, manage HIV-related complications, and mitigate ART toxicities in HIV patients, potentially yielding clinically meaningful improvements in life quality and longevity. However, the limited number of available studies highlights an imperative for well-designed multicenter trials with low risk of bias to detect realistic and meaningful effect sizes for integrated CM-ART therapeutic approaches.

## Data Availability

The original contributions presented in the study are included in the article/Supplementary Material, further inquiries can be directed to the corresponding authors.
