# Supplementary material for: The effect of Chinese medicine therapeutics on HIV/AIDS: a systematic review and network meta-analysis
Source: Front Reprod Health. 2025 Nov 4;7:1689063. doi: 10.3389/frph.2025.1689063 (PMC12623377; doi:10.3389/frph.2025.1689063)
Supplement: Supplementary file 1 [file Table1.docx]

Search strategy

| TableⅠ. PubMed | |
| --- | --- |
| No | Search items |
| #1 | "Medicine, Chinese Traditional"[Mesh] |
| #2 | ((((((((((((((Medicine, Chinese Traditional[Title/Abstract]) OR (Zhong Yi Xue[Title/Abstract])) OR (Chung I Hsueh[Title/Abstract])) OR (Hsueh, Chung I[Title/Abstract])) OR (Traditional Medicine, Chinese[Title/Abstract])) OR (Chinese Traditional Medicine[Title/Abstract])) OR (Traditional Chinese Medicine[Title/Abstract])) OR (Chinese Medicine, Traditional[Title/Abstract])) OR (Traditional Tongue Diagnosis[Title/Abstract])) OR (Tongue Diagnoses, Traditional[Title/Abstract])) OR (Tongue Diagnosis, Traditional[Title/Abstract])) OR (Traditional Tongue Diagnoses[Title/Abstract])) OR (Traditional Tongue Assessment[Title/Abstract])) OR (Tongue Assessment, Traditional[Title/Abstract])) OR (Traditional Tongue Assessments[Title/Abstract]) |
| #3 | #1 OR #2 |
| #4 | "Drugs, Chinese Herbal"[Mesh] |
| #5 | ((((((Drugs, Chinese Herbal[Title/Abstract]) OR (Chinese Drugs, Plant[Title/Abstract])) OR (Chinese Herbal Drugs[Title/Abstract])) OR (Herbal Drugs, Chinese[Title/Abstract])) OR (Plant Extracts, Chinese[Title/Abstract])) OR (Chinese Plant Extracts[Title/Abstract])) OR (Chinese Plant Extracts[Title/Abstract]) |
| #6 | #4 OR #5 |
| #7 | "Acupuncture"[Mesh] |
| #8 | (Acupuncture[Title/Abstract]) OR (Pharmacopuncture[Title/Abstract]) |
| #9 | #7 OR #8 |
| #10 | "Acupuncture Therapy"[Mesh] |
| #11 | ((((((((((Acupuncture Therapy[Title/Abstract]) OR (Acupuncture Treatment[Title/Abstract])) OR (Acupuncture Treatments[Title/Abstract])) OR (Treatment, Acupuncture[Title/Abstract])) OR (Therapy, Acupuncture[Title/Abstract])) OR (Pharmacoacupuncture Treatment[Title/Abstract])) OR (Treatment, Pharmacoacupuncture[Title/Abstract])) OR (Pharmacoacupuncture Therapy[Title/Abstract])) OR (Therapy, Pharmacoacupuncture[Title/Abstract])) OR (Acupotomy[Title/Abstract])) OR (Acupotomies[Title/Abstract]) |
| #12 | #10 OR #11 |
| #13 | "Moxibustion"[Mesh] |
| #14 | (Moxibustion[Title/Abstract]) OR (Moxabustion[Title/Abstract]) |
| #15 | #13 OR #14 |
| #16 | "Acupuncture Points"[Mesh] |
| #17 | (((((Acupuncture Points[Title/Abstract]) OR (Acupuncture Point[Title/Abstract])) OR (Point, Acupuncture[Title/Abstract])) OR (Points, Acupuncture[Title/Abstract])) OR (Acupoints[Title/Abstract])) OR (Acupoint[Title/Abstract]) |
| #18 | #16 OR #17 |
| #19 | "Meridians"[Mesh] |
| #20 | ((((Meridians[Title/Abstract]) OR (Jing Luo[Title/Abstract])) OR (Luo, Jing[Title/Abstract])) OR (Jingluo[Title/Abstract])) OR (Ching Lo[Title/Abstract]) |
| #21 | #19 OR #20 |
| #22 | "Massage"[Mesh] |
| #23 | ((((((((Massage[Title/Abstract]) OR (Zone Therapy[Title/Abstract])) OR (Therapies, Zone[Title/Abstract])) OR (Zone Therapies[Title/Abstract])) OR (Therapy, Zone[Title/Abstract])) OR (Massage Therapy[Title/Abstract])) OR (Massage Therapies[Title/Abstract])) OR (Therapies, Massage[Title/Abstract])) OR (Therapy, Massage[Title/Abstract]) |
| #24 | #22 OR #23 |
| #25 | "Musculoskeletal Manipulations"[Mesh] |
| #26 | (((((((((((((((((((Musculoskeletal Manipulations[Title/Abstract]) OR (Manipulations, Musculoskeletal[Title/Abstract])) OR (Craniosacral Massage[Title/Abstract])) OR (Massage, Craniosacral[Title/Abstract])) OR (Reflexology[Title/Abstract])) OR (Manipulation Therapy[Title/Abstract])) OR (Therapy, Manipulation[Title/Abstract])) OR (Manipulation Therapies[Title/Abstract])) OR (Therapies, Manipulation[Title/Abstract])) OR (Manipulative Therapies[Title/Abstract])) OR (Manipulative Therapy[Title/Abstract])) OR (Therapies, Manipulative[Title/Abstract])) OR (Therapy, Manipulative[Title/Abstract])) OR (Manual Therapies[Title/Abstract])) OR (Manual Therapy[Title/Abstract])) OR (Therapies, Manual[Title/Abstract])) OR (Therapy, Manual[Title/Abstract])) OR (Bodywork[Title/Abstract])) OR (Bodyworks[Title/Abstract])) OR (Rolfing[Title/Abstract]) |
| #27 | #25 OR #26 |
| #28 | "Cupping Therapy"[Mesh] |
| #29 | (((((Cupping Therapy[Title/Abstract]) OR (Cupping Therapies[Title/Abstract])) OR (Therapy, Cupping[Title/Abstract])) OR (Cupping Treatment[Title/Abstract])) OR (Cupping Treatments[Title/Abstract])) OR (Treatment, Cupping[Title/Abstract]) |
| #30 | #28 OR #29 |
| #31 | "Hyperthermia, Induced"[Mesh] |
| #32 | ((((((((Hyperthermia, Induced[Title/Abstract]) OR (Thermotherapy[Title/Abstract])) OR (Therapy, Fever[Title/Abstract])) OR (Fever Therapy[Title/Abstract])) OR (Induced Hyperthermia[Title/Abstract])) OR (Therapeutic Hyperthermia[Title/Abstract])) OR (Hyperthermia, Therapeutic[Title/Abstract])) OR (Hyperthermia, Local[Title/Abstract])) OR (Local Hyperthermia[Title/Abstract]) |
| #33 | #31 OR#32 |
| #34 | #3 OR #6 OR #9 OR #12 OR #15 OR #18 OR #21 OR #24 OR #27 OR #30 OR #33 |
| #35 | "HIV"[Mesh] |
| #36 | (((((((((((((((((((((((((HIV[Title/Abstract]) OR (HTLV-III[Title/Abstract])) OR (Human Immunodeficiency Virus[Title/Abstract])) OR (Immunodeficiency Virus, Human[Title/Abstract])) OR (Immunodeficiency Viruses, Human[Title/Abstract])) OR (Virus, Human Immunodeficiency[Title/Abstract])) OR (Viruses, Human Immunodeficiency[Title/Abstract])) OR (Human Immunodeficiency Viruses[Title/Abstract])) OR (Human T Cell Lymphotropic Virus Type III[Title/Abstract])) OR (Human T-Cell Lymphotropic Virus Type III[Title/Abstract])) OR (Human T-Cell Leukemia Virus Type III[Title/Abstract])) OR (Human T Cell Leukemia Virus Type III[Title/Abstract])) OR (LAV-HTLV-III[Title/Abstract])) OR (Lymphadenopathy-Associated Virus[Title/Abstract])) OR (Lymphadenopathy Associated Virus[Title/Abstract])) OR (Lymphadenopathy-Associated Viruses[Title/Abstract])) OR (Viruses, Lymphadenopathy-Associated[Title/Abstract])) OR (Virus, Lymphadenopathy-Associated[Title/Abstract])) OR (Human T Lymphotropic Virus Type III[Title/Abstract])) OR (Human T-Lymphotropic Virus Type III[Title/Abstract])) OR (AIDS Virus[Title/Abstract])) OR (AIDS Viruses[Title/Abstract])) OR (Virus, AIDS[Title/Abstract])) OR (Viruses, AIDS[Title/Abstract])) OR (Acquired Immune Deficiency Syndrome Virus[Title/Abstract])) OR (Acquired Immunodeficiency Syndrome Virus[Title/Abstract]) |
| #37 | #35 OR #36 |
| #38 | "Acquired Immunodeficiency Syndrome"[Mesh] |
| #39 | (((((((((((((((Acquired Immunodeficiency Syndrome[Title/Abstract]) OR (AIDS[Title/Abstract])) OR (Immunodeficiency Syndrome, Acquired[Title/Abstract])) OR (Acquired Immunodeficiency Syndromes[Title/Abstract])) OR (Immunodeficiency Syndromes, Acquired[Title/Abstract])) OR (Syndrome, Acquired Immunodeficiency[Title/Abstract])) OR (Syndromes, Acquired Immunodeficiency[Title/Abstract])) OR (Acquired Immune Deficiency Syndrome[Title/Abstract])) OR (Acquired Immuno-Deficiency Syndrome[Title/Abstract])) OR (Acquired Immuno Deficiency Syndrome[Title/Abstract])) OR (Acquired Immuno-Deficiency Syndromes[Title/Abstract])) OR (Immuno-Deficiency Syndrome, Acquired[Title/Abstract])) OR (Immuno-Deficiency Syndromes, Acquired[Title/Abstract])) OR (Syndrome, Acquired Immuno-Deficiency[Title/Abstract])) OR (Syndromes, Acquired Immuno-Deficiency[Title/Abstract])) OR (Immunologic Deficiency Syndrome, Acquired[Title/Abstract]) |
| #40 | #38 OR #39 |
| #41 | #37 OR #40 |
| #42 | "Randomized Controlled Trial" [Publication Type] |
| #43 | Randomized Controlled Trial[MeSH Major Topic] |
| #44 | Randomized Controlled Trial[Title/Abstract] |
| #45 | #42 OR #43 OR #44 |
| #46 | "Randomized Controlled Trials as Topic"[Mesh] |
| #47 | (((Randomized Controlled Trials as Topic[Title/Abstract]) OR (Clinical Trials, Randomized[Title/Abstract])) OR (Trials, Randomized Clinical[Title/Abstract])) OR (Controlled Clinical Trials, Randomized[Title/Abstract]) |
| #48 | #46 OR #47 |
| #49 | "Observational Study" [Publication Type] |
| #50 | #50((Observational Study[Title/Abstract]) OR (Natural Experiment Study[Title/Abstract])) OR (Natural Experiment[Title/Abstract])) OR (Naturalistic Observation Study[Title/Abstract]) |
| #51 | #51=#49 OR #50 |
| #52 | #52=”Observational Studies as Topic"[Mesh] |
| #53 | #53=((((Observational Studies as Topic[Title/Abstract]) OR (Observational Study as Topic[Title/Abstract])) OR (Natural Experiments as Topic[Title/Abstract])) OR (Natural Experiment as Topic[Title/Abstract])) OR (Naturalistic Observation Studies as Topic[Title/Abstract])) OR (Naturalistic Observation Study as Topic[Title/Abstract]) |
| #54 | #52 OR #53 |
| #55 | "Cohort Studies"[Mesh] |
| #56 | (((((((((((((((((((((((((((((((((Cohort Studies[Title/Abstract]) OR (Cohort Study[Title/Abstract])) OR (Studies, Cohort[Title/Abstract])) OR (Study, Cohort[Title/Abstract])) OR (Studies, Concurrent[Title/Abstract])) OR (Concurrent Study[Title/Abstract])) OR (Study, Concurrent[Title/Abstract])) OR (Concurrent Studies[Title/Abstract])) OR (Closed Cohort Studies[Title/Abstract])) OR (Cohort Studies, Closed[Title/Abstract])) OR (Closed Cohort Study[Title/Abstract])) OR (Cohort Study, Closed[Title/Abstract])) OR (Study, Closed Cohort[Title/Abstract])) OR (Studies, Closed Cohort[Title/Abstract])) OR (Historical Cohort Studies[Title/Abstract])) OR (Cohort Studies, Historical[Title/Abstract])) OR (Cohort Study, Historical[Title/Abstract])) OR (Historical Cohort Study[Title/Abstract])) OR (Study, Historical Cohort[Title/Abstract])) OR (Studies, Historical Cohort[Title/Abstract])) OR (Incidence Studies[Title/Abstract])) OR (Incidence Study[Title/Abstract])) OR (Studies, Incidence[Title/Abstract])) OR (Study, Incidence[Title/Abstract])) OR (Analysis, Cohort[Title/Abstract])) OR (Analyses, Cohort[Title/Abstract])) OR (Cohort Analyses[Title/Abstract])) OR (Cohort Analysis[Title/Abstract])) OR (Birth Cohort Studies[Title/Abstract])) OR (Birth Cohort Study[Title/Abstract])) OR (Cohort Studies, Birth[Title/Abstract])) OR (Cohort Study, Birth[Title/Abstract])) OR (Studies, Birth Cohort[Title/Abstract])) OR (Study, Birth Cohort[Title/Abstract]) |
| #57 | #55 OR #56 |
| #58 | "Systematic Review" [Publication Type] |
| #59 | (Review, Systematic[Publication Type]) OR (Umbrella Review[Publication Type]) |
| #60 | ((Systematic Review[Title/Abstract]) OR (Review, Systematic[Title/Abstract])) OR (Umbrella Review[Title/Abstract]) |
| #61 | #58 OR #59 OR #60 |
| #62 | "Systematic Reviews as Topic"[Mesh] |
| #63 | (((Systematic Reviews as Topic[Title/Abstract]) OR (Reviews Systematic as Topic[Title/Abstract])) OR (Systematic Review as Topic[Title/Abstract])) OR (Umbrella Reviews as Topic[Title/Abstract]) |
| #64 | #62 OR #63 |
| #65 | "Meta-Analysis" [Publication Type] |
| #66 | Meta-Analysis[MeSH Major Topic] |
| #67 | Meta-Analysis[Title/Abstract] |
| #68 | #65 OR #66 OR #67 |
| #69 | "Meta-Analysis as Topic"[Mesh] |
| #70 | (((((((Meta-Analysis as Topic[Title/Abstract]) OR (Meta Analysis as Topic[Title/Abstract])) OR (Clinical Trial Overviews[Title/Abstract])) OR (Clinical Trial Overview[Title/Abstract])) OR (Overview, Clinical Trial[Title/Abstract])) OR (Data Pooling[Title/Abstract])) OR (Data Poolings[Title/Abstract])) OR (Overviews, Clinical Trial[Title/Abstract]) |
| #71 | #69 OR #70 |
| #72 | #45 OR #48 OR #51 OR #54 OR #57 OR #61 OR #64 OR #68 OR #71 |
| #73 | #34 AND #41 AND #72 |

The search strategies for the Cochrane Library and the Web of Science database are aligned with those of PubMed.

| TableⅡ. Embase | |
| --- | --- |
| No | Search items |
| #1 | chinese medicine':ab,ti OR 'chinese herbal medicine':ab,ti OR 'chinese traditional medicine':ab,ti OR 'medicine, chinese traditional':ab,ti OR 'traditional chinese medicine':ab,ti |
| #2 | chinese medicine':ab,ti OR 'chinese herbal medicine':ab,ti OR 'chinese traditional medicine':ab,ti OR 'medicine, chinese traditional':ab,ti OR 'traditional chinese medicine':ab,ti |
| #3 | #1 OR #2 |
| #4 | herbaceous agent'/exp |
| #5 | drugs, chinese herbal':ab,ti OR 'herbaceous drug':ab,ti OR 'herbaceous plant':ab,ti OR 'herbaceous substance':ab,ti OR 'herbal agent':ab,ti OR 'herbal drug':ab,ti OR 'herbal material':ab,ti OR 'herbal medicinal product':ab,ti OR 'herbal preparation':ab,ti OR 'herbaceous agent':ab,ti |
| #6 | #4 OR #5 |
| #7 | acupuncture'/exp |
| **#8** | acupuncture therapy':ab,ti OR 'shonishin':ab,ti OR 'acupuncture':ab,ti  #9 =#7 OR #8 |
| #9 | #7 OR #8 |
| #10 | moxibustion'/exp |
| #11 | moxibustion':ab,ti |
| #12 | #10 OR #11 |
| #13 | acupuncture point'/exp |
| #14 | acupuncture point':ab,ti OR 'acu-point':ab,ti OR 'acupoint':ab,ti OR 'acupoints':ab,ti OR 'acupuncture points':ab,ti OR 'point, acupuncture':ab,ti |
| #15 | #13 OR #14 |
| #16 | body meridian'/exp |
| #17 | body meridian':ab,ti OR 'jing mai':ab,ti OR 'jingmai':ab,ti OR (meridian:ab,ti AND 'chinese medicine':ab,ti) OR 'meridians':ab,ti |
| #18 | #16 OR #17 |
| #19 | massage'/exp |
| #20 | massage':ab,ti OR 'sports massage':ab,ti OR 'massotherapy':ab,ti OR 'masso-therapy':ab,ti OR 'massage therapy':ab,ti |
| #21 | #19 OR #20 |
| #22 | cupping therapy'/exp |
| #23 | cupping therapy':ab,ti OR 'vacuum cupping':ab,ti OR 'suction cupping':ab,ti OR 'moving cupping':ab,ti OR 'flash cupping':ab,ti OR 'fire cupping':ab,ti OR 'cupping treatment':ab,ti OR 'cupping manipulation':ab,ti OR (cupping:ab,ti AND therapy:ab,ti) |
| #24 | #22 OR #23 |
| #25 | thermotherapy'/exp |
| #26 | thermotherapy':ab,ti OR 'thermal therapy':ab,ti OR 'therapy, heat':ab,ti OR 'infrared therapy':ab,ti OR 'infra red therapy':ab,ti OR 'induced hyperthermia':ab,ti OR 'hyperthermic treatment':ab,ti OR 'hyperthermic therapy':ab,ti OR 'hyperthermia, induced':ab,ti OR 'heat therapy':ab,ti OR 'dry heat therapy':ab,ti OR 'artificial hyperthermia':ab,ti OR 'ammotherapy':ab,ti |
| #27 | #25 OR #26 |
| #28 | ‘musculoskeletal manipulation’/exp |
| #29 | musculoskeletal manipulations ':ab,ti OR ' musculoskeletal manipulation ':ab,ti |
| #30 | #28 OR #29 |
| #31 | #3 OR #6 OR #9 OR #12 OR #15 OR #18 OR #21 OR #24 OR #27 OR #30 |
| #32 | human immunodeficiency virus'/exp |
| #33 | human immunodeficiency virus':ab,ti OR 'virus, lymphadenopathy associated':ab,ti OR 'lymphadenopathy associated virus':ab,ti OR 'lymphadenopathy associated retrovirus':ab,ti OR (lav:ab,ti AND aids:ab,ti) OR 'lav':ab,ti OR 'immunodeficiency associated virus':ab,ti OR 'human immuno deficiency virus':ab,ti OR 'hiv':ab,ti OR 'aids virus':ab,ti OR 'aids related virus':ab,ti OR 'aids associated virus':ab,ti OR 'aids associated retrovirus':ab,ti OR 'aids associated lentivirus':ab,ti |
| #34 | #32 OR #33 |
| #35 | acquired immune deficiency syndrome'/exp |
| #36 | acquired immune deficiency syndrome':ab,ti OR 'immunodeficiency, acquired':ab,ti OR 'human immunodeficiency virus infection/acquired immunodeficiency syndrome':ab,ti OR 'human immune deficiency virus/acquired immune deficiency syndrome':ab,ti OR 'hiv/aids':ab,ti OR 'aquired immunodeficiency syndrome':ab,ti OR 'aquired immune deficiency syndrome':ab,ti OR 'aids':ab,ti OR 'acquired immunodeficiency virus syndrome':ab,ti OR 'acquired immunodeficiency syndrome':ab,ti OR 'acquired immunodeficiency disease syndrome':ab,ti OR 'acquired immuno-deficiency syndrome':ab,ti OR 'acquired immune deficiency disease syndrome':ab,ti OR 'acquired human immunodeficiency syndrome':ab,ti |
| #37 | #35 OR 36# |
| #38 | #34 OR #37 |
| #39 | randomized controlled trial'/exp |
| #40 | randomized controlled trial':ab,ti OR 'trial, randomized controlled':ab,ti OR 'randomized controlled study':ab,ti OR 'randomised controlled trial':ab,ti OR 'randomised controlled study':ab,ti OR 'controlled trial, randomized':ab,ti |
| #41 | #39 OR #40 |
| **#42** | observational study'/exp |
| #43 | observational study':ab,ti OR 'observational study as topic':ab,ti OR 'observational studies as topic':ab,ti OR 'observational studies':ab,ti OR 'observation study':ab,ti OR 'observation studies':ab,ti OR 'nonexperimental study':ab,ti OR 'nonexperimental studies':ab,ti OR 'non experimental study':ab,ti OR 'non experimental studies':ab,ti |
| #44 | #37 OR #38 |
| #45 | cohort analysis'/exp |
| #46 | cohort analysis':ab,ti OR 'fertility, cohort':ab,ti OR 'cohort study':ab,ti OR 'cohort studies':ab,ti OR 'cohort life cycle':ab,ti OR 'cohort fertility':ab,ti OR 'analysis, cohort':ab,ti |
| #47 | #45 OR #46 |
| #48 | systematic review'/exp |
| #49 | systematic review':ab,ti OR 'review, systematic':ab,ti |
| #50 | meta analysis'/exp |
| #51 | meta analysis':ab,ti OR 'metaanalysis':ab,ti OR 'meta-analysis':ab,ti OR 'analysis, meta':ab,ti |
| #52 | #48 OR #4 OR #50 OR #51 |
| #51 | #41 OR #44 OR #47 OR #52 |
| #52 | #31 AND #38 AND #48 |
