# Supplementary figures and images for: The effect of Chinese medicine therapeutics on HIV/AIDS: a systematic review and network meta-analysis
Source: Front Reprod Health. 2025 Nov 4;7:1689063. doi: 10.3389/frph.2025.1689063 (PMC12623377; doi:10.3389/frph.2025.1689063)

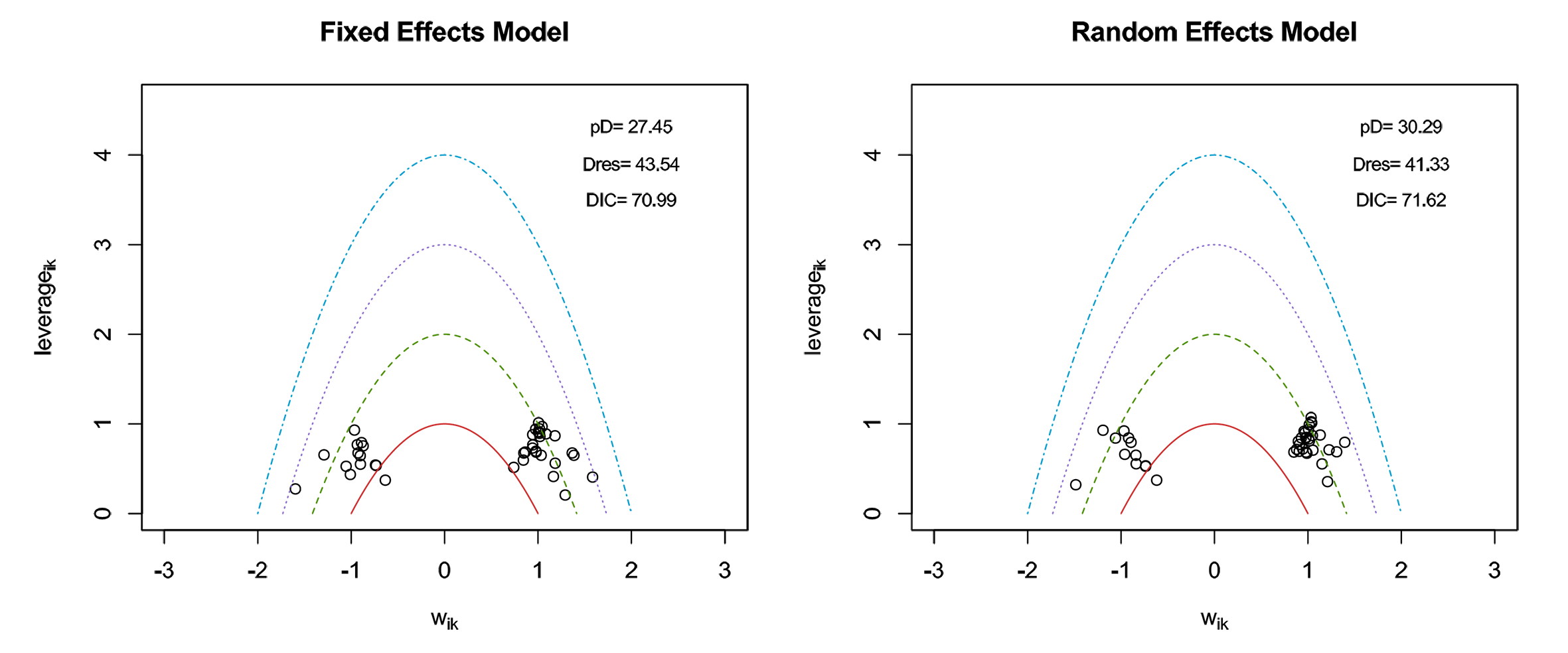

Supplement: Supplementary file 3 [file Image1.tif]

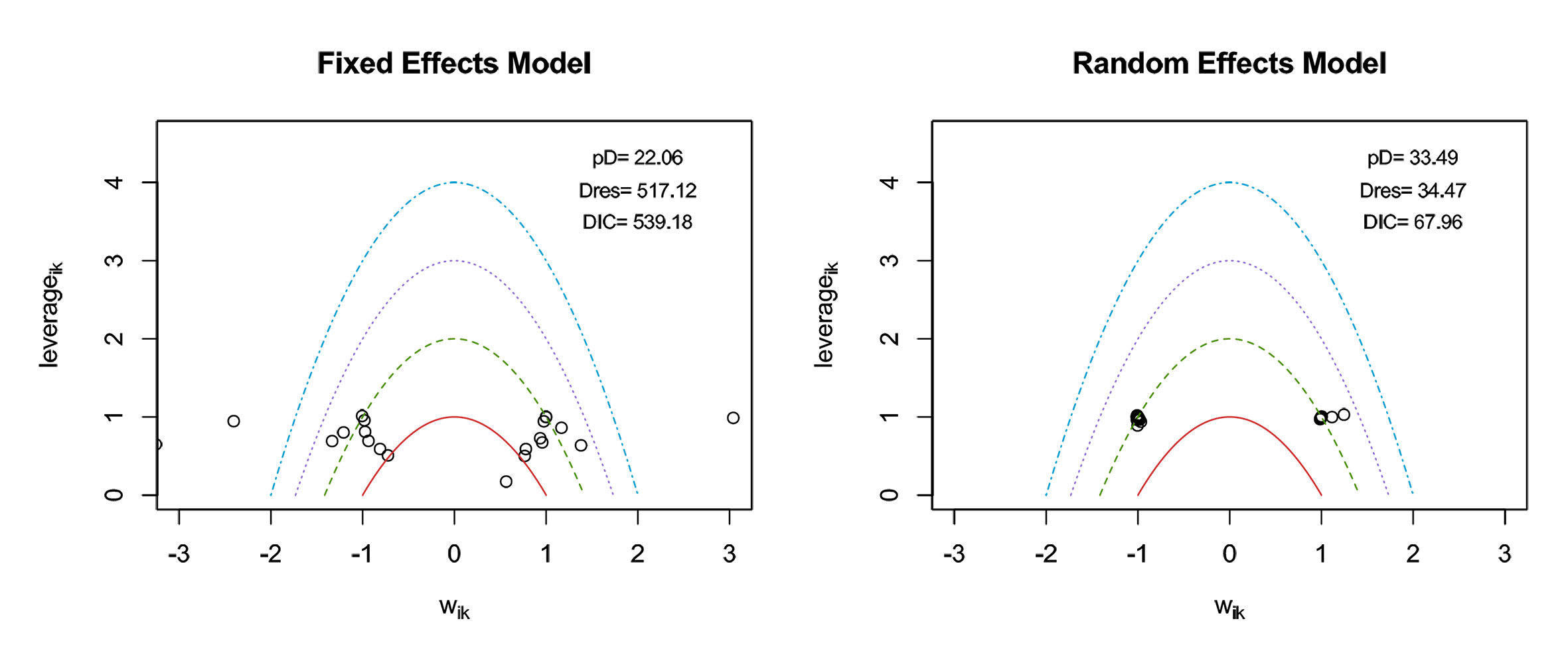

Supplement: Supplementary file 4 [file Image2.tif]

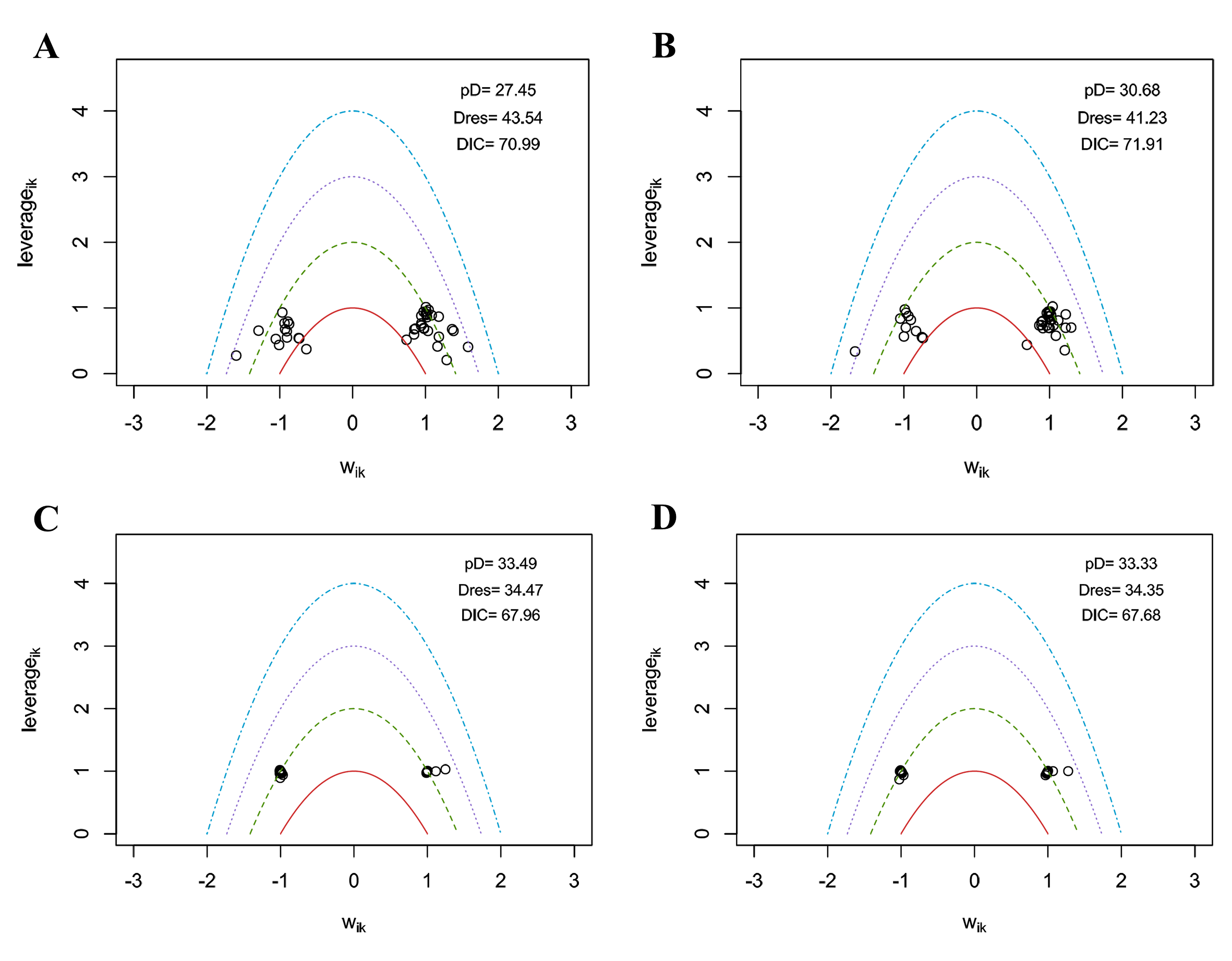

Supplement: Supplementary file 5 [file Image3.tif]

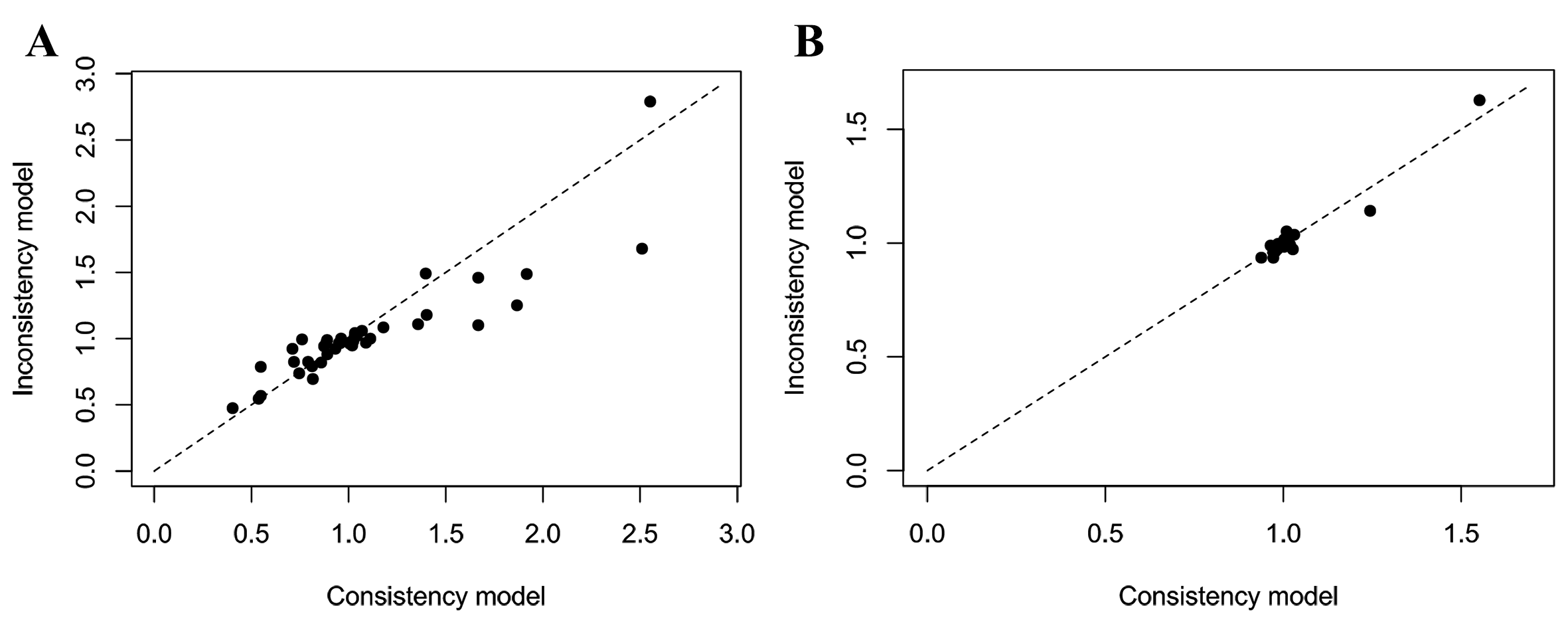

Supplement: Supplementary file 6 [file Image4.tif]
